# Hepatorenal Syndrome with Cirrhotic Cardiomyopathy: Case Report and Literature Review

**DOI:** 10.1155/2015/573513

**Published:** 2015-03-22

**Authors:** Luis Mocarzel, Pedro Lanzieri, Juliana Nascimento, Clara Peixoto, Mário Ribeiro, Evandro Mesquita

**Affiliations:** ^1^Department of Internal Medicine, Hospital Universitário Antônio Pedro (HUAP), Universidade Federal Fluminense (UFF), Rua Marquês de Paraná 303, 7° Andar, Centro, 24033-900 Niterói, RJ, Brazil; ^2^Department of Echocardiography, Hospital Universitário Antônio Pedro (HUAP), Universidade Federal Fluminense (UFF), Rua Marquês de Paraná 303, 7° Andar, Centro, 24033-900 Niterói, RJ, Brazil; ^3^Department of Cardiology, Hospital Universitário Antônio Pedro (HUAP), Universidade Federal Fluminense (UFF), Rua Marquês de Paraná 303, 7° Andar, Centro, 24033-900 Niterói, RJ, Brazil

## Abstract

The hepatorenal syndrome (HRS) is defined as a potentially reversible kidney failure in patients with cirrhosis and ascites. An association of HRS and cirrhotic cardiomyopathy has been reported recently, but there are no result studies about the use of positive inotropes as part of the acute phase treatment. We report the case of a patient diagnosed with HRS, with high levels of NT pro-BNP, but with normal ejection fraction of the left ventricle, which showed abnormalities in systolic function through speckle tracking in echocardiography, reversible after the infusion of dobutamine. The patient showed clinical and laboratory improvement of his renal function after the infusion of dobutamine. Clinical studies are needed on HRS therapeutic approach taking into account the myocardial dysfunction as a major contributing factor to renal dysfunction.

## 1. Introduction

The hepatorenal syndrome (HRS) is defined as a potentially reversible kidney failure in patients with liver cirrhosis and ascites. In its physiopathology, hyperactivity of the sympathetic and renin-angiotensin-aldosterone systems (RAAS), elevation of nitric oxide, and systemic vasodilatation are determinant factors [1]. Recently, a cardiomyopathic component associated with HRS has been reported [2]. HRS is classified into two types. In type I, there is a rapid elevation of serum creatinine, at least twice the normal range, which reflects a reduction of creatinine clearance of 50%, usually reaching levels of up to 2.5 mg/dL. HRS type II has a more insidious and less aggressive course, and its hallmark is ascites refractory to diuretics. HRS type I generally courses with oliguria (less than 400–500 mL/day) and is secondary to infections or metabolic disturbances. About half of the patients usually respond to the therapies currently used [3].

Cirrhotic cardiomyopathy (CMP) is now a well-established condition, defined as the presence of blunted ventricular response to stress, in cirrhotic patients, with a raised basal cardiac output accompanied by systolic, diastolic, electrophysiological, structural, histological, and biochemical changes [4, 5]. Recent studies have stated myocardial dysfunction in cirrhosis as a contributing factor, or even a precipitant, of HRS [2].

Current pharmacological therapies for HRS are based on the administration of human albumin in association with vasoconstrictors like noradrenaline, terlipressine, midodrine, and octreotide [3]. There are no solid papers determining the use of positive inotropes as part of the treatment of HRS patients.

We report a clinical case of a patient with HRS type I, with high levels of natriuretic propeptide (NT pro-BNP) but with normal left ventricle ejection fraction (LVEF) on echocardiogram, who presented abnormalities in his systolic function detected through speckle tracking echocardiography (STE), which proved to be reversible by the infusion of dobutamine. The patient had clinical and laboratory improvement of his kidney function after the use of the inotrope.

## 2. Case Presentation

A 63-year-old white male, born in the Northeast of Brazil and now living in Rio de Janeiro, presents a history of hepatic cirrhosis diagnosed nine years before, related to alcohol and hepatitis C virus infection, with previous admissions due to decompensation of cirrhosis. At the time he was on propranolol 20 mg t.i.d., omeprazole 40 mg s.i.d., spironolactone 150 mg s.i.d., and furosemide 40 mg s.i.d.

He was admitted to the emergency room with a three-day history of fever, progressive worsening dyspnea up to orthopnea, and abdominal distention with a diffuse pain. There was no alteration in his mental status or bowel movements. He had no previous history of nephropathy or heart disease.

A general physical exam revealed tachycardia of 100 beats per minute and tachypnea with respiratory effort and peripheral saturation of 94% in room air. His extremities were cold and his blood pressure was 90/70 mmHg with no postural fall. There were clinical signs of malnutrition, pallor, and voluminous ascites, with 69 k of body weight. In his respiratory examination, vesicular sounds and vocal fremitus on the right hemithorax were reduced. Cardiovascular exam and venous jugular pressure were normal. Abdomen showed tense ascites, with collateral circulations and peritoneal irritation without splenomegaly. There was neither a peripheral edema nor signs of hepatic encephalopathy. Laboratory tests showed a hematocrit of 27%, platelets of 104 × 10^3^/*μ*L, and white blood count of 6.600 × 10^3^/*μ*L (6% bands). Serum creatinine was 0.88 mg/dL and blood urea nitrogen (BUN) was 15 mmol/L. Prothrombin activity time was 16.4 seconds, albumin, 2 g/dL, and total bilirubin, 2.2 mg/dL (1.7 mgL/dL of direct fraction), Child-Pugh score C, 10 points, and Meld score, 15 points. Urine exam was positive for 3-4 leukocytes/mm^3^ and 2-3 red blood cells/mm^3^, with a protein-to-creatinine ratio in spot urine of 935 mg/g. Urine culture was negative.

Electrocardiogram revealed a sinus rhythm with normal waves morphology and corrected QT interval (QTc) of 460 msec for Bazett's formula.

Upper endoscopy showed large esophageal varices, erosive gastritis, and congestive gastropathy.

Spontaneous bacterial peritonitis was confirmed by a diagnostic and therapeutic paracentesis, and the fluid had a turbid appearance, with 600 leukocytes/*μ*L (50% of polymorphonuclear) and with albumin of 3 g/L, which was diagnostic of spontaneous bacterial peritonitis. The patient was started on ceftriaxone and because his clinical condition got worse, the scheme was changed to meropenem, with improvement of the infectious status. Abdominal fluid was indicative of intraperitoneal infection, and the clinical improvement after antibiotics supported this diagnosis, but blood cultures were negative.

On the fourth day after the antibiotic replacement, there was a reduction of the urinary output and a rise of BUN and creatinine. He was then prescribed albumin and noradrenaline at 8 *μ*g/kg/hour. Serum creatinine rose to 1.81 mg/dL. After three days, the patient continued to exhibit a urinary output lower than 200 mL/24 h, despite therapeutic measures, with a creatinine rise of up to 2.63 mg/dL. Because of this poor response, the NT pro-BNP was measured, 444 pg/dL (normal value: 135 pg/dL), suggesting a possible cardiomyopathic component associated. The therapeutic decision was to start dobutamine on 2.5 mcg/kg/minute, even in the absence of an echocardiographic study, which was unavailable at that time. The patient recovered diuresis 12 hours after the inotrope was started. Naturally, as a positive inotrope was started, as adjunctive therapy to the usual measures (albumin and noradrenaline infusion), the beta-blocker was temporarily held.

**Figure 1:**
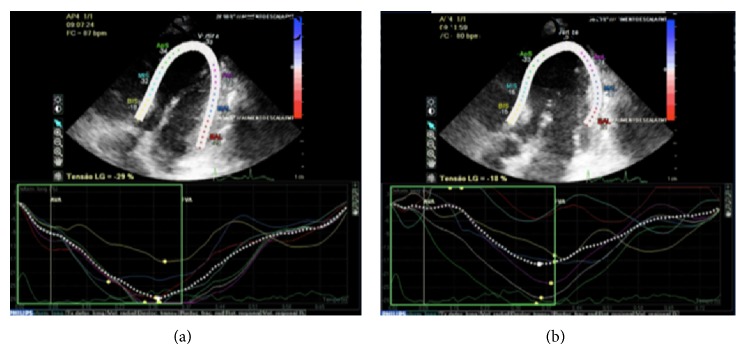
Speckle tracking echocardiogram with Doppler. Comparison between images during and after dobutamine infusion ((a) and (b)), showing differences in systolic global strain (29% and 18%, resp.).

After 24 hours of dobutamine infusion, an echocardiogram was done and showed an ejection fraction of 62.3% (Simpson's method) and a global longitudinal strain of 29%. Dobutamine was then withheld, and his condition was reevaluated after 30 minutes, with a reduction of the systolic global strain to 18% and the ejection fraction to 51.8%. After dobutamine infusion was restarted, there was a normalization of the parameters, as shown in Figure 1.

On the following five days, the urinary output progressively increased, with a reduction of serum creatinine to 1.29 mg/dL, when the dobutamine was withheld (Table 1).

The patient was discharged after fifteen days of the intervention, in association to normal urine output and serum creatinine at 1.25 mg/dL.

## 3. Discussion

Hemodynamic abnormalities in cirrhosis were described more than half a century ago by Kowalski and Abelmann [6]: pronounced arterial vasodilation with a reduction of peripheral vascular resistance, which activates autonomous nervous system and RAAS in order to maintain peripheral perfusion. This hyperdynamic state is directly dependent on the inotropic and chronotropic capacities of the myocardium so that the cardiac output remains stable [7].

In the last decade, cellular and functional changes in the heart of cirrhotic patients have been characterized, which suggest a cardiac dysfunction in the context of hepatic cirrhosis. These studies confirm the cardiomyopathic cirrhosis hypothesis, which denotes a condition of suboptimal cardiac response to stress, diastolic dysfunction, and electromechanical abnormalities [8–10]. These changes are described in about 40–50% of cirrhotic patients, regardless of the etiology of the liver disease [11–13].

The cardiomyocyte has membrane receptors which lead to energy production through cyclic AMP (cAMP) pathway. This function is depressed by different mechanisms present in stress situations: desensibilization and downregulation of beta-adrenergic receptors; reduced response to muscarinic receptors M2 and M3; dysfunction in K^+^ channels and in Na^+^/Ca^2+^ change channels; superexpression of endocanabinoid receptors; decrease of G protein; rise of tumor necrosis factor and nitric oxide. All these changes have as common pathway the decrease of energy production through cAMP, with consequent cardiomyocyte contractility impairment [7]. Cardiac dysfunction may negatively affect cirrhotic patients' prognosis, interfere in survival rates, and induce the development of other complications like HRS or acute heart failure during procedures such as liver transplants or transjugular intrahepatic portosystemic shunt implants (TIPS) [13].

Electrocardiographical changes most related to cirrhotic CMP are QTc interval prolongation in about half of the patients and electromechanical coupling defects. Correlations between the severity of liver disease and the presence of changes in electrocardiogram at rest have been found. [2, 5, 11] In the reported case, there was a prolonged QTc.

NT pro-BNP is an important biomarker of cardiac dysfunction from different etiologies, released by cardiomyocyte in response to high ventricle filling pressures [14]. This prohormone has diagnostic and prognostic values, but few papers have evaluated its clinical impact in patients with cirrhosis. Studies have shown a correlation between BNP and NT pro-BNP, the severity of liver disease, and the degree of systolic or diastolic cardiac dysfunction [15].

Echocardiography studies cardiac function in noninvasive parameters through LVEF, which was normal in this patient. The echocardiographic findings most commonly described in cirrhotic CMP are contractility impairment, diastolic dysfunction, and increase in left atrial volume, besides its correlation with portopulmonary hypertension and intrapulmonary shunts [16]. Recently, new echocardiography techniques, especially STE, have helped an early recognition of myocardial dysfunction, even if the LVEF is normal [17]. These findings may point to a hidden CMP, which can be proven by STE and contributed to a favorable clinical outcome of this patient. The use of dobutamine in the treatment of acute heart failure is widely accepted and recommended according to the American Heart Association guidelines [18]. Studies with dobutamine stress in cirrhotic patients are scarce and they generally describe changes in cardiac output, but with no increase in hepatic perfusion [19–21]. Cardiac response to inotropic stimulus may superpose to defects in beta-adrenergic signalizing pathway which are described in cirrhotic CMP.

The prognosis of HRS varies according to its type and is generally better in type I, when an intervention in the cause of the acute kidney decompensation may be possible [1]. Myocardial dysfunction in liver cirrhosis is a possible precipitating factor of HRS and, therefore, a potential therapeutic target [1, 3].

In the event of clinical and laboratory diagnosis of HRS refractory to the usual treatment in a patient with high NT pro-BNP and no history of cardiopathy, cirrhotic CMP was suspected as an important factor of HRS, since all the available measures for treatment of the HRS had already been tried and, at that point, dobutamine was started.

Studies have shown a growing importance of cardiac dysfunction as a factor that may worsen HRS prognosis [2, 4, 5], which supports the need for pharmacological intervention in this case, even though there was no clear recommendation for the specific therapeutic measures in this patient. The results of this case suggest that, after the standard therapy of HRS described in the literature, it may be important to consider the myocardial dysfunction as a relevant trigger and perpetrator of HRS.

It is recognized that sepsis is one of the most known intrinsic factors of HRS and circulatory dysfunction in cirrhotic patients. Cardiomyopathy aggravates hepatorenal syndrome, which supports the need for pharmacological intervention to improve cardiac function of these patients.

In the past, dobutamine and dopamine were used to be given for HRS. Due to lack of significant positive results, they are not recommended anymore. Besides treatment of underlying conditions and improvement of liver function, approach to therapy attempts to reverse the acute kidney injury associated with HRS [5]. If continuous monitoring is available, current recommendation is treatment with an intravenous infusion of norepinephrine at 0.5 to 3 mg/hr, aiming to raise the mean arterial pressure by 10 mmHg, and albumin is given for at least two days as an intravenous bolus (1 g/kg per day, up to 100 g). Vasopressin at 0.01 units/min titrated may be an alternative. If the patient is not in the ICU, options are terlipressin as an intravenous bolus (1 to 2 mg every four to six hours) in association with albumin or a combination of octreotide (subcutaneously 100 to 200 mcg three times daily or continuous intravenous infusion at 50 mcg/hr), midodrine (starting at 7.5 mg three times a day, up to 45 mg daily), and albumin [1–3].

The present case describes a patient with no history of cardiopathy who develops HRS type I in the context of spontaneous bacterial peritonitis and progresses to oliguria. Due to lack of response to usual therapeutic measures to HRS and high NT pro-BNP levels, a CMP component was inferred and dobutamine was started. An echo revealed a responsive cardiac dysfunction which, in association with clinical and laboratory improvement, points towards a hidden cardiomyopathy that, when treated, may hold up highly risky procedures such as TIPS and hemodialysis, since liver transplantation is rather inaccessible [13].

This index case suggests that clinical studies are needed to evaluate therapeutic approach with inotropes in HRS, considering the myocardial dysfunction as an important contributing factor to kidney dysfunction in HRS type I [22].

## Figures and Tables

**Table 1:** Chronologic evolution of serum creatinine and urinary output, in relation to the therapeutic interventions.

Days in hospital/intervention	Creatinine (mg/dL)	Urine output (mL/day)
01	0.72	>1000
16/**albumin/noradrenaline**	0.83	600
18	1.81	0
19	2.48	0
20/**added dobutamine**	2.63	0
21	2.13	900
22	1.56	1200
23	1.44	1200

## References

[1] Bonder A., Botero M. L., Cardenas A. (2014). Current therapies for hepatorenal syndrome. *Current Hepatitis Reports*.

[2] Wiese S., Hove J. D., Bendtsen F., Møller S. (2013). Cirrhotic cardiomyopathy: pathogenesis and clinical relevance. *Nature Reviews Gastroenterology & Hepatology*.

[3] Arroyo V., Ginès P., Gerbes A. L. (1996). Definition and diagnostic criteria of refractory ascites and hepatorenal syndrome in cirrhosis. *Hepatology*.

[4] Zardi E. M., Abbate A., Zardi D. M. (2010). Cirrhotic cardiomyopathy. *Journal of the American College of Cardiology*.

[5] Páll A., Czifra A., Vitális Z., Papp M., Paragh G., Szabó Z. (2014). Pathophysiological and clinical approach to cirrhotic cardiomyopathy. *Journal of Gastrointestinal and Liver Diseases*.

[6] Kowalski H. J., Abelmann W. H. (1953). The cardiac output at rest in Laennec's cirrhosis. *The Journal of Clinical Investigation*.

[7] Chayanupatkul M., Liangpunsakul S. (2014). Cirrhotic cardiomyopathy: review of pathophysiology and treatment. *Hepatology International*.

[8] Møller S., Bernardi M. (2013). Interactions of the heart and the liver. *European Heart Journal*.

[9] Møller S., Henriksen J. H. (2002). Cirrhotic cardiomyopathy: a pathophysiological review of circulatory dysfunction in liver disease. *Heart*.

[10] Møller S., Henriksen J. H. (2008). Cardiovascular complications of cirrhosis. *Gut*.

[11] Arroyo V., García-Martinez R., Salvatella X. (2014). Human serum albumin, systemic inflammation and cirrhosis. *Journal of Hepatology*.

[12] Páll A., Czifra A., Vitális Z., Papp M., Paragh G., Szabó Z. (2014). Pathophysiological and clinical approach to cirrhotic cardiomyopathy. *Journal of Gastrointestinal and Liver Diseases*.

[13] Møller S., Hove J. D., Dixen U., Bendtsen F. (2013). New insights into cirrhotic cardiomyopathy. *International Journal of Cardiology*.

[14] Iwanaga Y., Nishi I., Furuichi S. (2006). B-type natriuretic peptide strongly reflects diastolic wall stress in patients with chronic heart failure: Comparison between systolic and diastolic heart failure. *Journal of the American College of Cardiology*.

[15] Padillo J., Rioja P., Muñoz-Villanueva M. C. (2010). BNP as marker of heart dysfunction in patients with liver cirrhosis. *European Journal of Gastroenterology and Hepatology*.

[16] Mota V. G., Filho B. M. (2013). EcoDopplercardiografia na Doença Hepática Crônica: Revisão Sistemática. *Arquivos Brasileiros de Cardiologia*.

[17] Kazankov K., Holland-Fischer P., Andersen N. H. (2011). Resting myocardial dysfunction in cirrhosis quantified by tissue Doppler imaging. *Liver International*.

[18] Yancy W. C., Jessup M., Bozkurt B. (2013). Guideline for the management of heart failure. *Circulation*.

[19] Caramelo C., Fernandez-Munoz D., Santos J. C. (1986). Effect of volume expansion on hemodynamics, capillary permeability and renal function in conscious, cirrhotic rats. *Hepatology*.

[20] Mikulic E., Muñoz C., Puntoni L. E., Lebrec D. (1983). Hemodynamic effects of dobutamine in patients with alcoholic cirrhosis. *Clinical Pharmacology & Therapeutics*.

[21] Kim M. Y., Baik S. K., Won C. S. (2010). Dobutamine stress echocardiography for evaluating cirrhotic cardiomyopathy in liver cirrhosis. *The Korean Journal of Hepatology*.

[22] Nodari S., Palazzuoli A. (2011). Current treatment in acute and chronic cardio-renal syndrome. *Heart Failure Reviews*.

